# Hyperkalemia in heart failure: Foe or friend?

**DOI:** 10.1002/clc.23392

**Published:** 2020-05-23

**Authors:** Amina Rakisheva, Maria Marketou, Anna Klimenko, Tatyana Troyanova‐Shchutskaia, Panos Vardas

**Affiliations:** ^1^ Department of Cardiology Scientific Institution of Cardiology and Internal Diseases Almaty Kazakhstan; ^2^ Department of Cardiology Heraklion University Hospital Crete Greece; ^3^ RUDN University Moscow Russian Federation; ^4^ Department of Cardiology Scientific and Practical Center of Cardiology Minsk Belarus; ^5^ Department of Cardiology Heart Sector, Hygeia Hospitals Group Athens Greece

**Keywords:** angiotensin, heart failure

## Abstract

Hyperkalemia is a frequent and sometimes life‐threatening condition that may be associated with arrhythmia and cardiac dysfunction in patients with heart failure (HF). High potassium levels in HF represent both a direct risk for cardiovascular complication and an indirect biomarker of the severity of the underlying disease, reflecting neurohormonal activation and renal dysfunction. Evaluating the prevalence and significance of hyperkalemia in HF patients is essential for optimizing the use of potassium sparing agents, such the renin–angiotensin–aldosterone system inhibitors (RAASi) or angiotensin receptor‐neprilysin inhibitors and mineralocorticoid receptor antagonists, which represent a well‐established cornerstone and life‐saving therapy. In this review we discuss recent findings and current concepts related to the epidemiology, pathological mechanisms and implications of hyperkalemia, as well as novel therapeutic approaches to counteract it in patients with HF. The balance between optimizing life‐saving potassium sparing medication and minimizing hyperkalemia‐associated risk is much needed in patients with HF. Although older potassium‐binding agents are associated with serious adverse events, novel potassium‐binding drugs are effective in lowering potassium levels and are generally well tolerated. Novel potassium‐binding drugs, such as patiromer and sodium zirconium cyclosilicate, may help to optimize therapy in HF and achieve guideline‐recommended doses. Hyperkalemia is common in HF patients and is associated with a poorer prognosis and an increased risk of cardiovascular complications: Contrariwise, “moderate” potassium levels go with a better prognosis, while the emergence of new drugs, potassium binders, could allow target doses of RAASi to be achieved.

## 1 INTRODUCTION

Heart failure (HF) is a syndrome with high morbidity and mortality that has acquired pandemic dimensions, affecting more than 38 million people worldwide.^1^In addition, the progressive nature of HF makes it a leading cause of hospitalization, due to the deleterious effects of sustained neurohormonal activation and comorbidities.^2^ Electrolytic disturbances, most usually of potassium levels, very often accompany HF, are a hindrance to the optimization of medication and impose a further burden on the patient. Since potassium plays a major role in cardiac excitability and arrhythmias, dyskalemia is an important clinical problem that is associated with significant life‐threatening complications.^3^, ^4^ Although the definition is not consistent always, hyperkalemia is generally defined as a serum potassium level >5.0 to 5.5 mEq/L and can be further classified as mild (5.6‐6.0 mEq/L), moderate (6.1‐7.0 mEq/L), and severe (>7.0 mEq/L). Potassium secretion is highly dependent on renin‐angiotensin‐aldosterone system (RAAS) activity and renal perfusion, as well as sodium availability to the distal nephron. Hyperkalemia represents both a direct risk for cardiovascular morbidity and a potential biomarker of adverse prognosis and disease severity. Patients with HF are at particularly high risk for hyperkalemia, which is likely to reflect some medical comorbidity, especially renal dysfunction. In addition, it is one of the most common factors that impede the optimum pharmaceutical approach to HF, leading to underuse of renin‐angiotensin‐aldosterone system inhibitors (RAASi) and mineralocorticoid receptor antagonists (MRAs), which are the drugs that offer the most help and have changed the outcomes of HF patients.^5^ Unfortunately, some additional independent risk factors, such as diabetes mellitus (DM), advanced age and renal insufficiency, that exacerbate all‐cause and in‐hospital mortality and hospitalizations in HF patients, also further increase the risk of hyperkalemia. At the opposite end of the scale is hypokalemia, which can also be observed in HF patients and can increase the risk of mortality, mainly via arrhythmogenic complications.^6^, ^7^, ^8^ Thus, both high and low potassium levels are unacceptable in HF patients. Data from the literature highlight the need for careful potassium monitoring, especially in the presence of comorbidities, with special emphasis being placed on patients with a low estimated glomerular filtration rate (eGFR).^5^

This review aims to summarize the recent findings and the current concepts related to the epidemiology, pathological mechanisms, and implications of hyperkalemia, and to discuss novel therapeutic approaches in patients with HF. Although strategies of management are not evidence based, a deep investigation and understanding of these issues could form the basis for a better treatment of dyskalemia in those patients, and hence their optimum clinical approach.

## 2 REGULATION OF POTASSIUM HOMEOSTASIS

Potassium is a very important electrolyte for the maintenance of the physiologic cell function. Approximately 2% of total‐body K+ is in extracellular fluid, whereas 98% of K+ is in the intracellular compartment. Cellular Na^+^‐K^+^‐ATPase plays a key role in this homeostasis by pumping Na^+^ out of the cell and K^+^ into the cell and this process leads to a K^+^ gradient across the cell membrane which is vitally important for normal cellular function. The body has developed numerous mechanisms for maintaining serum K^+^ in a narrow range and kidney is the organ with the primary responsibility of this and adjustments in renal K^+^ excretion occur over several hours. Serum potassium homeostasis is usually preserved until GFR is reduced substantially.

There are several mechanisms regulating potassium secretion and potassium reabsorption. K^+^ is freely filtered across the glomerulus and then reabsorbed by the proximal tubule and thick ascending limb of the kidney. The shift in lumen potential from negative to positive in the proximal tubule provides a driving force for K^+^ reabsorption. In the thick ascending limb, K^+^ reabsorption is mediated mostly by the Na^+^‐K^+^‐2Cl^−^ cotransporter located on the apical membrane while a small proportion is reabsorpted through the paracellular pathway. Important factors for this regulation include luminal sodium delivery and flow rate, plasma potassium concentration, circulating aldosterone and arginine vasopressin, and acid‐base status. Αldosterone, angiotensin II, distal delivery of Na^+^ and water are key factors in the renal excretion of K^+^. Angiotensin II‐mediated stimulation of aldosterone secretion from the adrenal gland control renal blood flow and GFR and therefore potassium balance. Decreased renal excretion of K^+^ can be due to a decreased distal delivery of Na^+^, mineralocorticoid deficiency, and/or abnormal cortical collecting tubule function. In addition, catecholamines through β_2_‐stimulation regulate extracellular K^+^ concentration. K^+^ homeostasis mechanisms are also modulated in the gut and ranges according to potassium intake through diet.

## 3 INCIDENCE OF HYPERKALEMIA IN HF PATIENTS

In general, the incidence of hyperkalemia is 2% to 3%, but the latest data demonstrate a scatter up to 55% among hospitalized patients, reaching 73% in patients with CKD.^9^, ^10^, ^11^, ^12^ Observational data from a large Scandinavian cohort indicate that hypokalemia occurred in approximately 14% of patients in the clinical setting, with 33% recurrence, while hyperkalemia was less frequent: 7% of patients with 35.7% recurrence.^13^ In a recent Danish population‐based cohort study, almost four in 10 patients with HF developed hyperkalemia, and many patients have recurrent hyperkalemia episodes. Hyperkalemia risk was strongly associated with kidney function impairment and with severe clinical outcomes and death in HF.^14^ Even in well‐selected populations with careful electrolyte monitoring, such as those in the PARADIGM‐HF trial, the incidence of hyperkalemia with ACEi was 16% over a median follow‐up time of 27 months.^15^

It has also been shown that hyperkalemia is present in 9% of patients admitted for acute HF.^16^ One study showed that 10% to 38% of hospitalized patients who received an ACEi developed hyperkalemia during hospitalization and 10% of patients prescribed an ACEi developed severe hyperkalemia within 1 year of follow‐up.^12^, ^17^, ^18^ In another, which analyzed a large cohort of 19 194 patients with new‐onset HF in the UK, 11% developed hyperkalemia during a 4‐year follow‐up.^19^

However, apart from the high incidence of hyperkalemia in patients who have HF with reduced ejection fraction (HFrEF), it is also common in HF with preserved ejection fraction (HFpEF). Indeed it may be even more common in HFpEF than in HFrEF, since this population is older, with a high burden of comorbidities and kidney failure. The TOPCAT trial, which examined the impact of treatment with spironolactone on clinical outcomes in HFpEF, found an increased incidence of hyperkalemia in those patients.^20^ However, the TOPCAT findings showed that the cutoff of >5.0 mEq/L was not associated with an adverse prognosis, suggesting that the guidance provided in the trial to adjust study drug dosing only for sustained K+ values ≥5.5 mEq/L is reasonable in clinical practice.^20^The European Society of Cardiology Heart Failure Long‐Term Registry (ESC‐HF‐LT) has reported that the presence of hyperkalemia in chronic HF is 2.64%, reaching 4.44% in patients with acute HF.^21^ The discrepancies between the various studies are due not only to their different patient populations, but also to the different definitions of hyperkalemia used and the presence of the various risk factors mentioned above. Recently, Savarese et al. have shown that the risk of moderate or severe hyperkalemia was highest in HFpEF and HF with midrange ejection fraction, whereas risk of hypokalemia was highest in HFpEF. HF severity, low hemoglobin, COPD, baseline high and low potassium, and low eGFR were relevant predictors of dysK occurrence.^22^

The extent to which hyperkalemia is implicated in the morbidity of HF patients is difficult to assess, since it may be asymptomatic and may only be discovered through routine blood screening, or as a consequence of the condition leading to hospitalization (eg, infection, dehydration, or deteriorating kidney function), rather than causing the hospitalization itself. Patients at high risk for hyperkalemia are often those with disease states such as HF and CKD, who also stand to benefit most from RAASi. However, most clinical trials have excluded patients with advanced CKD, who are those at the highest risk for hyperkalemia; consequently, we lack evidence regarding the therapeutic management and long‐term outcomes in this population.

## 4 PROGNOSTIC IMPLICATIONS OF HYPERKALEMIA IN HF

Systemic hyperkalemia results in a shortening of the membrane action potential and altered conduction velocity. Initially, there is a decrease in the effective refractory period, but as hyperkalemia worsens increased K+ channel conductances can induce post‐repolarization refractoriness and prolongation of the effective refractory period. The electrocardiogram first demonstrates peaked T waves resulting from global action potential shortening. Subsequently, the P wave broadens and decreases in amplitude, eventually disappearing, and the QRS widens. Severe hyperkalemia (K+ >7.0 mmol/L) can lead to heart block, asystole, and ventricular tachycardia or fibrillation (VT/VF). Similarly, interstitial hyperkalemia during acute ischemia has all of the electrophysiological actions described above, which are exacerbated by concomitant hypoxia or acidosis. Interstitial hyperkalemia also promotes early repolarization in the ischemic subepicardium, inducing phase 2 re‐entry and VT/VF analogous to Brugada syndrome.^23^Apart from cardiac arrhythmias, potassium disorders can also result in muscle cramps, muscle weakness, rhabdomyolysis, and myoglobinuria.

Although serum potassium up to 5.5 mEq/L appears relatively safe in HF,^7^ many studies have pointed out the association between high serum potassium and adverse clinical outcomes in HF patients. It has been reported that the rate of in‐hospital death associated with hyperkalemia in HF patients reaches 0.39 per 1000 patients.^24^

A Spanish registry has shown that, in patients hospitalized for decompensated HF, admission hyperkalemia predicts a higher mid‐term risk for HF readmission and mortality, probably related to the significant higher risk of readmission.^25^

On the MEESSI‐AHF scale, hyperkalemia is a variable associated with a greater risk of 30‐day mortality.^26^Conversely, correction of hyperkalemia to normokalemia leads to a lower risk of all‐cause mortality during follow‐up.^27^

On the other hand, hyperkalemia has been associated with both increased and decreased mortality risk in various trials.^20^, ^28^, ^29^, ^30^ This largely depends on the clinical substrate, that is, whether hyperkalemia is coming from renal dysfunction or due to potassium sparing drugs. The latter is associated with improved prognosis in specific populations and avoidance of hypokalemia. This may explain why a high potassium level may be protective especially in the long term. In contrast, in critically ill patients and in patients with chronic kidney disease, high potassium levels are associated with high rates of arrhythmias and cardiac arrest. Notwithstanding, published studies demonstrating an association of mortality with hyperkalemia are largely limited to retrospective analyses and have no design to provide evidence of causation.

Hoss et al. suggested that spironolactone and loop diuretic therapy appear to be safe in patients with high‐normal potassium levels, HF and reduced renal function and are associated with an improved outcome.^29^

In a large, geographically diverse United States population receiving medical care, 27.6% had a potassium level <4.0 mEq/L, while 5.7% had ≥5.0 mEq/L. A U‐shaped association was revealed between serum potassium and mortality, with the lowest all‐cause mortality seen in controls with potassium values between 4.0 and <5.0 mEq/L.^28^ However, the effect on mortality was more severe for hypokalemia than for hyperkalemia, since the investigators observed 45.5% mortality with moderate‐to‐severe hypokalemia and a 35.7% death rate associated with hyperkalemia in those with HF.

After publication of the RALES trial,^31^ hospitalization for hyperkalemia in the Canadian health system rose significantly, and this was associated with an increased risk of death among those patients. A decline in eGFR exceeding 20% or 30% was independently associated with a more frequent occurrence of all clinical adverse events. In an EPHESUS post hoc analysis,^32^ four independent baseline predictors of hyperkalemia (defined as ≥6.0 mEq/L) were identified: baseline potassium greater than the median (4.3 mEq/L), baseline eGFR ≤60 mL/min per 1.73 m^2^, a history of diabetes mellitus, and previous use of antiarrhythmic agents. In the BIOSTAT‐CHF cohort, at baseline, hypokalemia (<3.5 mEq/L) was present in 6.9% and hyperkalemia (>5.0 mEq/L) in 8.0%.^33^ On the other hand, discontinuation of RAASi therapy exhibited a J‐shaped trend in association with serum potassium. Basnet et al., in a study of 2 660 609 patients who were discharged with a diagnosis of HF, reported that patients with hypokalemia during hospitalization had a greater mortality risk than those with hyperkalemia.^34^ Low serum potassium is known to increase the transmembrane resting potential of myocardial cells, resulting in increased excitability and leading to a higher risk of atrial fibrillation, Q‐T interval prolongation, torsade des pointes, ventricular arrhythmias, ventricular fibrillation, and sudden cardiac death. This might explain the fact that hypokalemia in HF has been associated with an increased risk of mortality, with hazard ratios ranging from 1.2 to 2.3.^7^, ^8^, ^35^

Notably, the Swedish HF registry, using real world data, showed that during the 1‐year follow‐up from baseline, 1.45 deaths per 1000 patient‐years occurred after hyperkalemia compared to 0.33 deaths that occurred in periods without or preceding hyperkalemia.^22^ The results were similar across the spectrum of HF. The risk of moderate or severe hyperkalemia or severe hypokalemia was the same and had the same prognostic impact regardless HF category. In contrast, the same study found that the incidence of hyperkalemia was not associated with increased hospitalizations.^22^ In addition, post hoc results from the TOPCAT‐Americas study, a large clinical trial with HFpEF patients indicate that among patients with eGFRs of <45 mL/min/1.73 m^2^, for every 100 patients treated with spironolactone, they would expect to prevent nine occurrences of the primary composite outcome but provoke 27 drug discontinuations because of hyperkalemia.^36^

## 5 PROTECTIVE EFFECTS OF POTASSIUM

Diets high in potassium have been associated with a lower incidence of hypertension and heart failure.^37^ Experimental data show that high potassium protects against hypertensive and sodium‐induced endothelial dysfunction, independently of blood pressure.^38^, ^39^, ^40^ In animal models, increasing plasma potassium improves endothelial function and arterial stiffness, reducing the rate of thrombosis on endothelial lesions.^39^, ^40^ Notably, high potassium levels have been shown to have protective effects against stroke and kidney disease in hypertensive rats.^41^

In general terms, serum potassium 4 to 4.9 mEq/L is optimal and 5 to 5.5 mEq/L appears relatively safe in HF.^7^ In a large cohort of patients with acute HF, patients with higher serum potassium were found to have a better diuretic response and low potassium was one of the strongest predictors of a poor diuretic response.^16^ It also appears that eGFR is lower in HF patients with higher potassium levels, which probably means that high potassium levels reflect worse renal function and may be a risk marker rather than a risk factor.^16^

In the EPHESUS trial, eplerenone also reduced the risk of hypokalemia, which was twice as large as the risk of serious hyperkalemia.^32^ Important findings from EMPHASIS‐HF suggest that the favorable effects of eplerenone on all‐cause death were independent of the incidence of hyperkalemia or worsening renal function.^42^, ^43^ On the other hand, a sub‐study from the RALES trial in chronic HF found that an increase in potassium levels after spironolactone treatment was not associated with an increase in mortality.^31^ In the RALES trial, the benefit of spironolactone was maintained in the setting of moderate hyperkalemia, and clinical outcomes with spironolactone were superior to those with placebo when potassium levels remained <6.0 mEq/L.^31^ In this trial, 13.5% and 40% of participants exhibited hyperkalemia when treated with 25 and 50 mg daily of spironolactone, indicating that hyperkalemia in HF is not always an ominous sign. Results from a recent, large, network‐wide analysis suggested that there was no difference in outcomes between patients with normal or mildly elevated potassium levels.^31^ Notably, serious hyperkalemia occurred in only 2% and 1% of patients in the spironolactone and placebo arms, respectively.^31^

## 6 HYPERKALEMIA IN LARGE CLINICAL TRIALS

The cornerstone of HF treatment is RAASi and MRAs and current HF guidelines^5^ recommend them for patients who have HFrEF unless they are contraindicated or not tolerated. There is evidence to show that these drugs can increase potassium levels from <2% to 5%‐10%, depending on the baseline characteristics.^3^, ^30^, ^44^ The smallest increase is observed among hypertensive patients without any risk factors, while the greatest is among patients with HF and CKD. Thus, HF treatment management always takes this kind of interaction into account and often limits the prescription of RAASi and its target dose titration. Apparently, most of them involved MRAs, whether spironolactone or eplerenone. Thus, RALES, EPHESUS, and EMPHASIS‐HF are three large randomized studies of MRAs in patients with HFrEF.^30^, ^31^, ^32^, ^45^ The ATHENA‐HF trial was also conducted to assess MRAs among patients with acute HF, regardless of ejection fraction.^46^ Another study in patients with HFrEF and NYHA II‐IV, PARADIGM‐HF, compared sacubitril/valsartan with enalapril.^15^ TOPCAT was the only study where HFpEF was a main inclusion criterion.^47^ A safety analysis of the abovementioned studies is presented in Table 1. In the RALES, EPHESUS, and TOPCAT studies hyperkalemia was identified when potassium level was >5.0 mmol/L and only the EMPHASIS‐HF trial identified hyperkalemia as a potassium level >4.5 mmol/L. Furthermore, the following stages of hyperkalemia were defined in this trial: mild (4.5 mmol/L), moderate (5.0 mmol/L), and severe (≥5.50 mmol/L).^30^, ^31^, ^41^, ^46^ Mineralocorticoids contribute to potassium loss and sodium absorption, while their antagonists have the reverse action and cause potassium absorption. Thus, the serum potassium level was higher among patients in the treatment group of MRA trials in comparison with placebo controls. However, MRAs improve prognosis despite leading to increased rates of hyperkalemia or hypokalemia.^30^ Apart from primary cardiovascular prevention of MRAs an additional advantage is the maintenance of potassium levels within normal range and the protection from hypokalemia.

**TABLE 1:** Hyper‐ and hypokalemia occurrence among patients in most important large randomized trials with potassium sparing agents in heart failure

Study name	Criteria of hyperkalemia used in large trials	Number of patients with hyperkalemia	Number of patients with hypokalemia
RALES^31^	*Mild* K^+^ 5.0‐5.4 mmol/L *Moderate* K^+^ 5.5 to 5.9 mmol/L *Severe* K^+^ ≥ 6.0 mmol/L	156 patients in spironolactone group and 47 in placebo	53 patients in spironolactone group and 136 in placebo
EPHESUS^32^	K^+^ >5.0 mmol/L	113 (3.4%) in eplerenone group and 66 (2.0%) placebo group	15 (0.5%) in eplerenone group and 49 (1.5%) placebo group
EMPHASIS‐HF^43^	Hyperkalemia variably defined as K^+^ >4.5, >5, or >5.5 mmol/L	158 patients (11.8%) in the eplerenone group and 96 patients (7.2%) in the placebo group (*P* < .001).	K <3.5 mmol/L was reported in 100 patients (7.5%) in the eplerenone group and 148 (11.0%) in the placebo group (*P* = .002)
TOPCAT^20^	K^+^ ≥ 5.0 mmol/L	18.7% in the spironolactone group vs 9.1% in the placebo group)	16.2% in the spironolactone group vs 22.9% in the placebo.
ATHENA‐HF^46^	Moderate >5.5 mmol/L; Severe >6.0 mmol/L	Only one patient in the group receiving usual care and 0 in the group taking high‐dose spironolactone experienced serum potassium levels between 5.5 and 5.9 mEq/L, and no one had a potassium concentration of >6.0 mEq/L during the 96 h of study treatment. Serious adverse events by 30 days were reported in 84 patients (47%) in the group receiving usual care and 79 patients (43%) taking high‐dose spironolactone (*P* = .47).	
PARADIGM‐HF^15^	K^+^ >5.5 mmol/L K^+^ ≥ 6.0 mmol/L	674 (16.1%) in sacubitril/ valsartan vs 727 (17.3%) in enalapril; *P* = .15 181 (4.3%) in sacubitril/ valsartan vs 236 (5.6%) in enalapril; *P* = .007	139 (3.31%) in sacubitril/valsartan vs 107 (2.53%) in enalapil

Although ATHENA‐HF was also an MRA trial, its design was different and focused on an evaluation of the standard therapy with spironolactone of 25 mg, or a higher dose of 100 mg, vs placebo.^46^There were only one event of high potassium level in the standard treatment arm and no hyperkalemia event in the group with a high spironolactone dose. The other study, PARADIGM‐HF, was conducted to compare two disease‐modifying drugs, sacubitril/valsartan and enalapril; since both of them contribute to hyperkalemia, serum potassium levels >5.5 mmol/L were registered in both groups. Nevertheless, a trend for higher potassium level (≥6.0 mmol/L) was identified in the enalapril arm (4.3% vs 5.6%; *Р* = .007).^15^

Hypokalemia was frequently identified among patients in the placebo group in the RALES, EMPHASIS‐HF, and EPHESUS trials, and was associated with increased mortality, especially when the serum potassium level was <3.5 mmol/L.^30^, ^31^, ^32^, ^42^ The TOPCAT trial compared spironolactone and placebo in patients with HFpEF, found an increase in potassium levels in the treatment arm and a significant decrease in the placebo arm.^47^There were no serious adverse effects in either group.^47^ Notably, a potassium level above 4.8 mmol/L has been related with increased mortality on clinical trials with HF patients and thus has been recommended by some researchers as the upper normal limit on HF.^28^

## 7 HYPERKALEMIA AS AN OBSTACLE TO OPTIMUM HF TREATMENT

Drug‐induced hyperkalemia is one of the most common causes of hyperkalemia in HF. Unfortunately, this fear results in clinicians' inertia who very often discontinue or down‐titrate medications that induce it. On the other hand, disease modifying drugs statistically reduce the risk of mortality in HF patients, and their adverse effect, such as hyperkalemia, contribute to an improvement of prognosis only when serum potassium levels show moderate elevation. Meanwhile, the development of hypokalemia worsens prognosis and increases mortality. The same effect was observed if the potassium level was over ≥6.0 mmol/L. Although mild hyperkalemia is not a clinical problem, given the risk of development of more severe hyperkalemia, serum potassium levels should be closely monitored inhigh‐risk patients. While in the era of trials such as RALES and EMPHASIS‐HF,^30^, ^42^ the rate of discontinuation is low in clinical practice, among new users of MRA therapy rates of discontinuation in the first year are high even with mild hyperkalemia. Of the patients who experience hyperkalemia, almost half discontinue the drug, with only a small percentage trying a reduced dose, and of those who discontinue the drug because of hyperkalemia, few restart.^48^ In a large retrospective clinical trial in patients with new onset HF, serum potassium concentrations outside 4.0 to 5.0 mmol/L were associated not only with elevated mortality risk, but also with increased likelihood of RAASi discontinuation.^49^

Although the management of hyperkalemia in these patient populations may include reduction or discontinuation of RAASi, this may have a negative impact on outcomes. Adverse cardiorenal outcomes have been observed to increase with RAASi dose reduction or discontinuation, particularly in cohorts with CKD and HF.^50^ Therefore, optimizing the therapeutic dosage of RAASi is encouraged, despite any difficulties in managing hyperkalemia. Due to the high prevalence of comorbidities among patients with HF, there is a risk that target doses of the disease‐modifying drugs cannot be achieved, reducing the effectiveness of the therapy.

Although the problem of hyperkalemia in these patients appears significant, one if its clear causes are the lack of sufficient control of potassium levels in primary care, which leads to the hyperkalemia rates described above, together with their complications. Nilsson et al. examined patients starting MRA therapy between 2007 and 2010 in Sweden and found that only 24% were adequately monitored at the recommended intervals.^51^ The prompt recognition of hyperkalemia usually leads to its treatment, improving the rates of optimum treatment in HF patients.

In general, HF patients with renal dysfunction, high potassium levels (>5.0 mEq/L) or bilateral renal stenosis might need a rechecking of potassium levels within 1 to 2 weeks after the initiation of RAASi and then every 3 to 5 months, while treatment may be stopped or the dose reduced when potassium is >5.5 mEq/L. Regarding MRAs in these patients, potassium is checked after 1 week, then 4, and then every 3 months; again, treatment is stopped or tapered when the potassium level reaches >5.5 mEq/L.

In every case, we should try to restart treatment. HF treatment guidelines do not encourage MRAs use in patients with an eGFR <30 mL/min per 1.73 m^2^ or serum potassium levels >5.0 mmol/L. In addition, it is important to instigate dietary counseling regarding potassium and reappraise concomitant medications (eg, potassium supplements, nonsteroidal anti‐inflammatory drugs, etc.) in those patients.

## 8 HYPERKALEMIA MANAGEMENT: FOCUS ON NEW TREATMENTS

**FIGURE 1:**
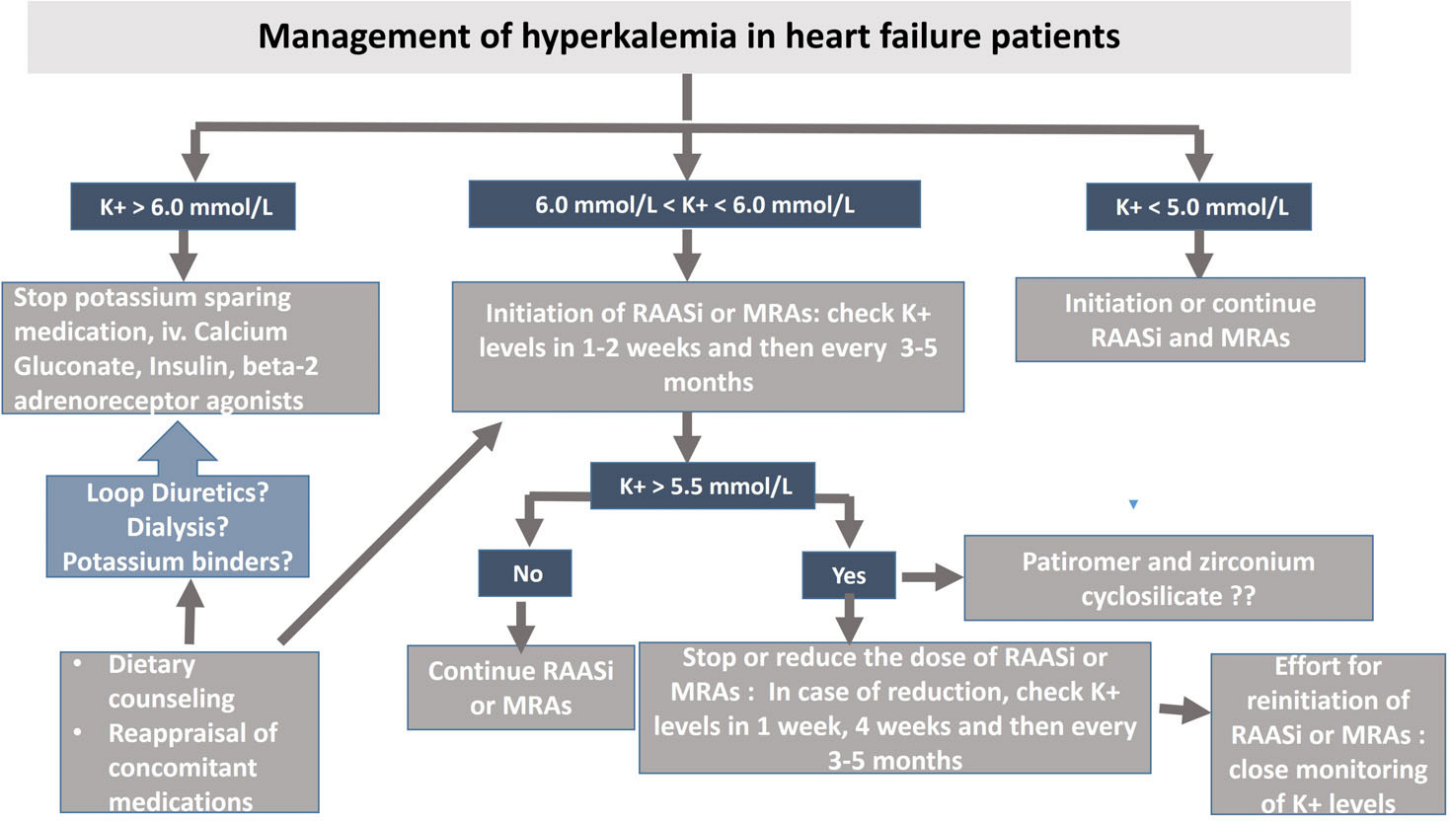
Steps in the therapeutic management of hyperkalemia in patients with heart failure. RAASi, renin angiotensin aldosterone system inhbitors; MRAs, mineralocorticoid receptor antagonists

The treatment of chronic hyperkalemia can be difficult and may be life‐threatening if left untreated. Potassium binders such as sodium polystyrene sulfonate is mainly used in patients with CKD and hyperkalemia. This substance is only suitable for short‐term treatment since its use is associated with hypernatremia, volume overload and gastrointestinal complications. A suggested management of chronic hyperkalemia in HF patients is presented in Figure 1. However, their use is associated with adverse effects, their efficacy is ascertain and their effects are transient. In this regard, the introduction of patiromer, acting at the level of the gastrointestinal tract, in particular in the colon lumen, is relevant,^20^, ^21^, ^23^, ^24^ may decrease serum potassium levels and enable treatment with RAASi and MRAs in significantly higher doses. Patiromer for oral suspension is a non‐absorbed, sodium‐free potassium binding polymer that exchanges calcium for potassium in the gastrointestinal tract. Patiromer beads are insoluble, have an average particle size of ≈100 μm and so are too large to be absorbed, excreted ≈24 to 48 h after intake and bind potassium predominantly in the lumen of the colon and this increases fecal potassium excretion, reducing levels of free potassium.^20^, ^21^, ^23^, ^24^ The mean reduction of serum potassium levels by patiromer in large clinical trials was in the range 0.45 to 1 mmol/L.^52^

Zirconium cyclosilicate (SCZ, former ZS‐9)—an inorganic cation that allows a thermodynamically favorable catching of potassium ions‐ represent another significant pharmacologic advancements in the treatment of hyperkalemia. More specifically SCZ is an oral, nonabsorbed, high‐capacity cation‐binding compound that selectively exchanges K^+^ for hydrogen and sodium ions throughout the gastrointestinal tract and has rapid onset of action. A single treatment with SCZ may reduce serum K^+^ levels at 1 h after the initial dose, results in a continued and sustained reduction in serum K^+^ levels for up to 48 h after the initial dose and has a favorable safety profile.^53^ The mean reduction of serum potassium levels by SCZ in large clinical trials was in the range 0.3 to 1.1 mmol/L.^52^

Table 2 summarizes the main studies whose aim was to evaluate the effect of potassium binding agents. An important inclusion criterion of the AMETHYST‐DN phase II study and the OPAL‐HK phase III study was the presence of a serum potassium level above 5.0 mEq/L, CKD and RAASi use.^54^, ^55^ Another patiromer study, PEARL‐HF, was conducted for patients with documented HF and a potassium level of 4.3 to 5.1 mEq/L.^56^ Additional inclusion criteria were either a reduction of eGFR to ≤60 mL/min/1.73 m^2^ while taking RAASi, or documented hyperkalemia that led to the cancellation of RAASi.

**TABLE 2:** Key inclusion criteria and results in indicative potassium binder trials

Study name	Number of patients	Agent	Mean follow‐up	Inclusion criteria	Results
AMETHYST‐DN^54^	324	Patiromer (4.2 g, 8.4 g or 12.6 g twice daily [mild hyperkalemia] or 8.4 g, 12.6 g, or 16.8 g twice daily [moderate hyperkalemia]).	52 weeks	eGFR:15 to <60 mL/min/1.73 m^2^ K^+^ >5.0 mEq/L Type 2 diabetes Receiving an ACEi, an ARB, or both for ≥28 days	Mild hyperkalemia: Mean K+ reduction −0.35 mEq/L for the 4.2 g, −0.51 mEq/L for the 8.4 g, −0.55 mEq/L for the 12.6 g Moderate hyperkalemia: the reduction was −0.87 mEq/L for the 8.4 g, −0.97 mEq/L for the 12.6 g, and −0.92 mEq/L for the 16.8 g hypokalemia (<3.5 mEq/L) occurred in 5.6% of patients
OPAL‐HK ^55^	243	Patiromer (8.4 or 16.8 g)	4 weeks	3rd or 4th stage of CKD eGFR −15 to 60 mL/min/1.73 m^2^; K^+^ 5.1 to 6.5 mmol/L stable dose of one or more RAASi for ≥28 days	Reduction of K+ levels −1.01 mmol/L vsplacebo (*P* < .001)
PEARL‐HF ^56^	120	Patiromer (15 g b.i.d. or placebo)	4 weeks	CHF a serum K^+^ 4.3‐5.1 mEq/L.In addition: (i) CKD [with (eGFR) of 60 mL/min] and were receiving one or more HF therapies (ACE‐Is, ARBs, beta‐blockers); or (ii) a documented history of hyper‐kalaemia that led to discontinuation of therapy with an AA, ACE‐I, ARB, or beta‐blocker within 6 months prior to the baseline visit.	Reduction of K+ levels −0.45 mmol/L vsplacebo (*P* < .001)
TOURMALINE^57^	112	Patiromer (8.4 g)	3 or 4 weeks	K^+^ >5.0 mmol/L; 67 receiving RAASi	From baseline to week 4, the mean (SE) change in serum potassium was −0.67 (0.08) mEq/L in patients taking RAASi and − 0.56 (0.10) mEq/L in patients not taking RAASi
HARMONIZEHF subgroup^58^	94	Sodium zirconium cyclosilicate (5, 10, or 15 g or placebo)	28 days	K^+^ >5.0 mmol/L; 60 receiving RAASi	Treatment maintained a lower potassium level (4.7 mmol/L, 4.5 mmol/L, and 4.4 mmol/L, respectively) than the placebo group and greater proportions of patients (83%, 89%, and 92%, respectively) maintained normokalaemia than placebo overall study population.
HARMONIZE‐Global Study^59^	248	Sodium zirconium cyclosilicate (thrice‐daily 10 g during a 48 h correction phase, patients achieving normokalaemia were randomized to once‐daily 5 g, 10 g, or placebo)	28 days	K^+^ >5.1 mmol/L	
Roger et al.^60^	751	Sodium zirconium cyclosilicate 10 g three times daily	365 days	K+ ≥5.1 mmol/L	100% and 95% with baseline eGFR <30 and ≥30 mL/min/1.73 m^2^ achieved normokalaemia
ZS‐005^61^	751	Sodium zirconium cyclosilicate 10 g three times daily for 24 to 72 h	365 days	K+ ≥5.1 mmol/L	mean serum potassium values ≤5.1 and ≤5.5 mmol/L were achieved by 99% of participants

Abbreviations: ACEi, angiotensin converting enzyme inhibitor; ARB, angiotensin receptor blocker.

Both AMETHYST‐DN and OPAL‐HK showed reductions of serum potassium level throughout the study, regardless of whether patients had chronic heart failure, or advanced CKD, DM, or hypertension.^54^, ^55^ The treatment period of PEARL‐HF showed a lower incidence of hyperkalemia in the patiromer group compared with the placebo group (7% vs 25%, respectively).^56^ Contrariwise, more patiromer‐treated patients developed hypokalemia, with a K^+^ value <3.5 mEq/L (6% vs 0%, respectively) and significantly more had a K^+^ value <4.0 mEq/L compared with the placebo group (47% and 10%, respectively). That led to an increase in the spironolactone dose in patients treated with patiromer compared with placebo patients (*P* = .019). In another open label study that included patients with baseline K^+^ < 5.0 mEq/L, patiromer was effective and generally well‐tolerated for hyperkalemia treatment, whether or not patients were taking RAAS inhibitors.^57^

The results from using SCZ were encouraging: the HARMONIZE trial showed that all three doses of SCZ were effective in lowering and maintaining normal potassium levels, including patients receiving RAASi therapy, who showed similar safety profiles.^58^ In HARMONIZE‐Global study, normokalemia achieved in a geographically and ethnically diverse population of outpatients with hyperkalemia.^59^ The most commonly reported AEs were oedema and constipation.^59^ SZC corrects hyperkalaemia and maintains normokalaemia among outpatients regardless of the CKD stage.^1^, ^60^

Unfortunately, these agents cannot be used as emergency treatment while the short duration of the above studies is a limitation in the management of chronic hyperlakemia. Although outcome studies are lacking, the use of potassium binders could play an important role in reaching target doses of RAASi by preventing the development of significant hyperkalemia. However, this hypothesis requires further research on combination treatment with potassium binders before guidelines can recommend their use in therapy.

## 9 CONCLUSIONS

Hyperkalemia is common in HF patients and is associated with a poorer prognosis and an increased risk of cardiovascular complications: this undoubtedly presents hyperkalemia as our “foe.” Contrariwise, “moderate” potassium levels go with a better prognosis, while the emergence of new drugs, potassium binders, could allow target doses of RAASi to be achieved, giving us an opportunity to think of hyperkalemia as a “friend.”

Although, important clinical trials^32^, ^42^, ^43^ in HF indicated that a potassium level of 5.0 to 5.5 mmol/L, according to the study design, does not offset the therapeutic benefits of HF treatment. Although, potassium levels >6.0 mEq/L have been associated with bradycardia, asystole, and sudden death, the exact modes of death related to hyperkalemia, is not clearly described in the large trials. Considering the risk of RAASi discontinuation,^49^ serum potassium concentrations between 4.0 and 5.0 mmol/L may be an ideal level. Dyskalemia incidence and related outcomes across epidemiological studies—specially in HFpEF is at present problematic and further studies are required to provide confidence as to the exact level of serum potassium below and above the normal range, which should be of concern and should trigger intervention. The balance between optimizing life‐saving potassium sparing medication and minimizing hyperkalemia‐associated risk is much needed in patients with HF.

## CONFLICT OF INTEREST

The authors declare no conflict of interest.
